# Better organized care via care pathways: A multicenter study

**DOI:** 10.1371/journal.pone.0180398

**Published:** 2017-07-03

**Authors:** Deborah Seys, Luk Bruyneel, Svin Deneckere, Seval Kul, Liz Van der Veken, Ruben van Zelm, Walter Sermeus, Massimiliano Panella, Kris Vanhaecht

**Affiliations:** 1 Leuven Institute for Healthcare Policy, KU Leuven - University of Leuven, Leuven, Belgium; 2 Department of Quality Management, UZ Leuven, Leuven, Belgium; 3 Medical Department, Delta Hospitals Roeselare, Roeselare, Belgium; 4 Department of Biostatistics, Faculty of Medicine, University of Gaziantep, Gaziantep, Turkey; 5 Q Consult, Utrecht, The Netherlands; 6 Department of Clinical and Experimental Medicine, University of Eastern Piedmont 'A. Avogadro', Novara, Italy; Public Library of Science, FRANCE

## Abstract

An increased need for efficiency and effectiveness in today’s healthcare system urges professionals to improve the organization of care. Care pathways are an important tool to achieve this. The overall aim of this study was to analyze if care pathways lead to better organization of care processes. For this, the Care Process Self-Evaluation tool (CPSET) was used to evaluate how healthcare professionals perceive the organization of care processes. Based on information from 2692 health care professionals gathered between November 2007 and October 2011 we audited 261 care processes in 108 organizations. Multilevel analysis was used to compare care processes without and with care pathways and analyze if care pathways led to better organization of care processes. A significant difference between care processes with and without care pathways was found. A care pathway in use led to significant better scores on the overall CPSET scale (p<0.001) and its subscales, “coordination of care” (p<0.001) and “follow-up of care” (p<0.001). Physicians had the highest score on the overall CPSET scale and the five subscales. Care processes organized by care pathways had a 2.6 times higher probability that the care process was well-organized. In around 75% of the cases a care pathway led to better organized care processes. Care processes supported by care pathways were better organized, but not all care pathways were well-organized. Managers can use care pathways to make healthcare professionals more aware of their role in the organization of the care process.

## Introduction

A large body of evidence demonstrates a large variation in aligning the organization of patient care with evidence based guidelines [1–5]. There is also a rather low level of agreement on what constitutes appropriate patient care. Quality deficiencies may result from the gap between guidelines and practice [6]. There is an increased pressure to improve the quality of care and organize it more efficiently. Healthcare quality is defined by the Institute of Medicine as “the extent to which health services provided to individuals and patient populations improve desired health outcomes. The care should be based on the strongest clinical evidence and provided in a technically and culturally competent manner with good communication and shared decision making” [7]. Low adherence to guidelines may contribute to preventable harm and suboptimal patient outcomes [8]. Care pathway implementation is a well-established strategy to standardize care. It reduces unnecessary complexity and the variation of care processes in and between organizations [9–11]. The European Pathway Association (E-P-A) defines a care pathway as “a complex intervention for the mutual decision making and organization of care for a well-defined group of patients during a well-defined period” [12]. Care pathways are associated with improved patient outcomes, team outcomes and better organized care processes. First, examples of improved patient outcomes include less post-operative complications and reduced length of stay [13–15]. It has also been found that the hospitalization costs were significantly lower when care pathways had been introduced [16]. Second, there are multiple positive effects on teams, including: better team communication, better documentation between professionals, better team relations and lower risk of burnout and task orientation [11, 13, 15, 17, 18]. When a care pathway is introduced, staff feel an urge to collaborate within the hospital and with primary care, which lead to better organized care processes [19]. Other examples of better care organization are standardization of care process, better documentation and communication with the patients and the healthcare professional, better follow-up of the care process and tasks are performed more confidently [15, 17, 20, 21].

There are still questions of what the effect is of care pathways on the organization of care. A Belgian study showed that care pathways had a positive impact on the organization of care, the coordination of care and the follow-up of care. One of the challenges was to improve the patient-focused organization, communication with patients and family, and collaboration with primary care [22]. One of the limitations of this study was that the care process was evaluated by only one medical doctor, one head nurse and one pathway facilitator immediately after validation of the tool. In practice, a team exists of more than these types of healthcare professionals [22].

The aims of this study are (1) to determine if care processes organized by care pathways are better perceived than care processes organized without care pathways. (2) To evaluate the extent to which team scores correlate for care processes with or without care pathways, and (3) to assess the sensitivity and specificity in predicting well-organized care processes.

## Materials and methods

### Sample and data collection

This study uses a cross-sectional multicenter convenience sample in Belgium and the Netherlands. Data were collected between November 2007 and October 2011 among 108 past or present members of the Belgian-Dutch Clinical Pathway Network (www.nkp.be) (Box 1). Most data were collected as part of the 10 day course organized by NKP. During this course, participants receive training on the concept and methodology of care pathways (www.nkp.be). All these organizations were given the opportunity to use the Care Process Self-Evaluation Tool (CPSET) to evaluate the quality of the organization of their care processes [23]. The team leader distributed the questionnaires to the physicians, nurses, allied health professionals, coordinator and other paramedics who were involved in that specific care process. The team members were asked to complete the questionnaire and return it to their team leader in a closed envelope. Returning a completed questionnaire implied consent to participate in this survey. This method of consent was approved by the ethics committee of the University Hospitals Leuven before the questionnaires were used in general practice. Anonymity and confidentiality were guaranteed. A total of 3378 questionnaires were completed over the four-year period. CPSET questionnaires with more than 10% missing values were excluded for further analysis. The statistical analysis included 2692 questionnaires from 87 Belgian organization and 21 organizations from the Netherlands. The included organizations are acute hospitals, psychiatric hospitals, specialized hospitals and primary care. A total of 261 audits of care processes were performed through these questionnaires. Almost all care processes (n = 259) were evaluated either before the implementation of a care pathway, during the development phase or after implementation. Two care processes were evaluated twice: one was evaluated before the implementation and during the development of their care pathway and the other was evaluated during the development and after implementation.

**Box 1**

The Belgian-Dutch Clinical Pathway Network (NKP) was launched in 2000 with eight hospitals. In 2013, NKP directly links 103 organizations in two countries (Belgium, the Netherlands) which actively develop, implement and evaluate clinical pathways. The activities can be organized in four groups: (1) academic support, e.g. workgroups for specific patient groups, (2) education on clinical pathways and related concepts, (3) research and (4) international collaboration. More details can be found on www.nkp.be.

### Measures

The questionnaire contained two sections. The first section included questions on type of profession, age, gender, involved patient group, if a care pathway (CP) was used or not, and if yes for how long. Care processes were categorized in: care processes with no care pathway (NCP), CP in development and CP in use. The second section of the questionnaire contained the CPSET. This is a tool to evaluate the quality of the organization of a care process. Content, face, construct and criterion validity and the reliability of this tool are excellent and have been described previously [23]. The CPSET contains 29-items, for which five subscales have previously been defined using factor analytic techniques [23]. These subscales are patient-focused organization (6 items), coordination of care (7 items), communication with patient and family (4 items), collaboration with primary care (3 items) and follow-up of care (9 items) (S1 Table). Each item is scored on a 10-point scale ranging from totally disagree (1) to totally agree (10) [23]. The CPSET is available in Dutch (original), English, French, Spanish and Norwegian [24]. The Dutch version that was used in this study has been extensively validated [23, 25].

### Statistical analysis

First, demographic characteristics for healthcare professionals involved in care processes without care pathways, care pathways under development and care pathways are given.

Secondly, to determine if care processes organized by care pathways are better perceived than care processes organized without care pathways (aim 1), overall and subscale scores are described and a two-level regression model was performed with respondents as first level and team as second level (random intercept). In our study, a team was defined as three or more healthcare professionals who work together on a care process. In this model we controlled for age and gender as both variables significantly influences CPSET scores [25]. As previous research showed that the perception regarding patient safety climate differed across professional groups [26], the findings are presented for all healthcare professionals jointly and separately. Statistical significance was defined as a p-value of <0.05.

Third, to evaluate the extent to which team scores correlate for care processes organized with or without care pathways (aim 2), the interclass correlation coefficient (ICC) and its 95% confidence interval (CI) were calculated to evaluate the within-cluster dependence.

Fourth, to assess the sensitivity and specificity in predicting well-organized care processes (aim 3), receiver operating characteristics (ROC) and area under the curve (AUC) were calculated for the five subscales and the overall CPSET score. These were calculated to identify a cut-off score, which make it possible to identify weakly and well-organized care process. A cut-off score of 0.65 was used for further analysis. To detect well-organized care processes, sensitivity, specificity, likelihood ratios (LRs) and odds ratios were calculated.

Statistical analysis were performed in SPSS 19 (http://www-01.ibm.com/support/docview.wss?uid=swg21476197), ICCs were calculated using the formula derived by Donner and Klar based on analysis of variance [27] and by using the multilevel package in R software (version 2.15.1).

## Results

### Demographic characteristics

Slightly less than half (48.9%; n = 1316) of the participants were aged 40 years or older, 68.3% (n = 1839) were female and about half were employed as a nurse (53.2%; n = 1431) (Table 1). Some teams included a project team facilitator for the care process. These are described by the term coordinator. The term “others” refers to patient care associates and unit service associates.

**Table 1:** Demographic characteristics.

Characteristics	NCP N (%) (N = 1025)	CP under development N (%) (N = 975)	CP N (%) (N = 692)
Age			
20–29	216 (21.1%)	188 (19.3%)	164 (23.7%)
30–39	282 (27.5%)	270 (27.7%)	207 (29.9%)
40–49	282 (27.5%)	316 (32.4%)	169 (24.4%)
50–59	209 (20.4%)	176 (18.1%)	127 (18.4%)
>60	25 (2.4%)	7 (0.7%)	5 (0.7%)
Unknown	11 (1.1%)	18 (1.8%)	20 (2.9%)
Gender			
Male	315 (30.7%)	320 (32.8%)	168 (24.3%)
Female	705 (68.8%)	646 (66.3%)	488 (70.5%)
Unknown	5 (0.5%)	9 (0.9%)	36 (5.2%)
Profession			
Nurse	537 (52.4%)	482 (49.4%)	412 (59.5%)
Physician	199 (19.4%)	175 (17.9%)	101 (14.6%)
Allied health professional	161 (15.7%)	175 (17.9%)	126 (18.2%)
Coordinator	55 (5.4%)	59 (6.1%)	15 (2.2%)
Others	73 (7.1%)	84 (8.6%)	38 (5.5%)
Average number of team members	10	8	15
Teams included	98	117	46

NCP: no care pathway

CP: care pathway

### Quality of organization of care

Descriptive analysis are displayed in Table 2. The three columns on the left show that compared with NCP and CP under development, mean scores on the overall CPSET and the five subscales were higher when CP were implemented. Also compared to other healthcare professionals, mean scores of physicians were highest for the overall CPSET for both NPC, CP under development and CP. This finding persisted for all subscales. The three columns on the right show the findings for the multilevel regression model. Findings show that mean scores on the overall CPSET are significantly higher when CP were implemented, compared to NCP and CP under development. This effect persisted for all types of healthcare professionals. The mean scores for the subscales measuring coordination of care and follow-up of care were also significantly higher when CP were implemented. Again this effect persisted for all healthcare professionals. For some subscales similar effects were found for certain types of healthcare professionals.

**Table 2:** Differences in care processes subscale CPSET.

				Estimates of regression model *		
	NCP average(SD)	CP under development average(SD)	CP average(SD)	CP versus NCP ß (SE)	CP versus CP in development ß (SE)	CP in development versus NCP ß (SE)
Overall CPSET	65.3 (13.0)	66.2 (11.4)	70.7 (11.9)	4.35 (1.15)***	4.01 (1.01)***	0.54 (0.92)
Nurse	63.7 (13.2)	65.1 (11.6)	68.3 (12.2)	2.97 (1.38)**	2.60 (1.25)**	0.45 (1.17)
Physician	68.8 (11.7)	69.0 (12.2)	75.7 (10.5)	6.60 (1.73)***	6.61 (1.62) ***	-0.34 (1.40)
Allied health professional	66.0 (14.5)	66.2 (10.6)	72.9 (10.8)	6.76 (2.13)**	5.90 (1.78)**	1.20 (1.89)
Coordinator	63.0 (9.0)	63.7 (9.2)	72.7 (7.0)	9.19 (2.75)**	9.33 (2.84)**	0.48 (1.71)
Others	67.3 (12.0)	68.0 (10.8)	74.0 (11.2)	8.68 (2.41)***	7.11 (2.46)**	1.57 (1.89)
Patient focused organization	72.9 (14.9)	73.4 (13.1)	76.1 (13.6)	1.68 (1.30)	2.23 (1.17)	-0.46 (1.05)
Nurse	71.6 (15.6)	73.2 (13.2)	74.6 (14.3)	0.69 (1.60)	0.76 (1.44)	-0.04 (1.35)
Physician	75.7 (13.6)	74.9 (14.1)	79.9 (12.0)	3.27 (1.95)	4.83 (1.74)**	-1.84 (1.59)
Allied health professional	72.3 (14.7)	72.1 (12.5)	76.3 (12.2)	3.59 (2.18)	3.65 (1.96)	-0.01 (1.95)
Coordinator	73.6 (12.6)	73.0 (10.8)	77.1 (7.6)	2.32 (3.49)	2.98 (3.26)	-0.05 (2.22)
Others	75.8 (13.2)	73.5 (12.3)	79.9 (14.8)	6.03 (2.73)**	7.17 (3.19)**	-1.59 (2.14)
Coordination of care	68.2 (14.5)	67.9 (12.6)	73.9 (12.4)	4.87 (1.27)***	5.76 (1.11)***	-0.69 (-0.67)
Nurse	67.7 (14.7)	67.6 (12.4)	72.8 (12.8)	3.67 (1.46)**	4.60 (1.29)***	-0.86 (1.24)
Physician	72.5 (12.6)	69.9 (13.6)	77.2 (12.0)	4.98 (1.87)**	7.63 (1.91)***	-2.69 (1.53)
Allied health professional	66.4 (16.1)	67.5 (12.8)	74.5 (11.8)	8.05 (2.45)**	6.32 (2.08)**	1.89 (2.18)
Coordinator	63.0 (11.5)	64.9 (11.5)	75.0 (7.9)	11.11 (3.57)**	9.78 (3.50)**	1.85 (2.26)
Others	68.2 (14.3)	68.4 (11.6)	75.8 (11.8)	10.03 (2.65)***	1.45 (2.82)**	0.61 (2.07)
Communication with patient and family	61.2 (18.3)	63.2 (16.8)	65.2 (17.1)	3.00 (1.64)	1.59 (1.45)	1.58 (1.45)
Nurse	57.3 (18.9)	59.9 (16.7)	61.3 (16.9)	1.37 (2.04)	0.26 (1.73)	1.38 (1.72)
Physician	67.7 (15.2)	70.7 (16.3)	73.8 (15.1)	4.78 (2.33)**	2.50 (2.34)	2.08 (1.88)
Allied health professional	63.9 (19.2)	63.1 (17.7)	69.4 (15.1)	6.44 (2.87)**	5.28 (2.65)**	1.51 (2.58)
Coordinator	60.8 (14.8)	60.9 (11.7)	69.5 (16.0)	6.87 (4.32)	8.93 (4.11)	-0.35 (2.68)
Others	66.5 (14.8)	67.5 (14.0)	69.5 (18.2)	5.41 (3.55)	2.64 (3.54)	2.07 (2.74)
Collaboration with primary care	65.8 (16.8)	66.6 (15.2)	68.7 (15.0)	2.53 (1.31)	2.14 (1.15)	0.66 (1.07)
Nurse	63.9 (16.6)	64.6 (15.8)	65.5 (14.4)	0.78 (1.66)	0.06 (1.54)	0.25 (1.43)
Physician	70.6 (17.0)	71.8 (15.6)	76.2 (14.4)	5.33 (2.25)**	4.80 (2.18)**	-0.06 (1.84)
Allied health professional	66.2 (17.0)	66.2 (13.5)	71.3 (15.0)	4.69 (2.40)	4.67 (2.02)**	0.44 (2.16)
Coordinator	63.6 (13.1)	64.3 (11.9)	75.6 (8.3)	12.50 (3.61)**	13.39 (3.27)**	0.12 (2.32)
Others	67.1 (17.6)	68.8 (13.8)	71.1 (14.5)	5.57 (3.33)	2.18 (2.86)	2.75 (2.62)
Follow-up of care	58.6 (17.2)	60.1 (17.0)	69.8 (14.4)	9.89 (1.47)***	8.63 (1.35)***	1.50 (1.20)
Nurse	58.5 (16.9)	60.3 (17.0)	68.1 (14.3)	8.67 (1.73)***	7.31 (1.66) ***	1.42 (1.48)
Physician	57.3 (17.4)	58.2 (19.6)	70.9 (16.1)	13.95 (2.51)***	12.01 (2.57)***	1.57 (2.07)
Allied health professional	61.3 (17.9)	62.1 (15.3)	73.2 (13.6)	10.90 (2.68)***	10.44 (2.45)***	1.16 (2.40)
Coordinator	54.2 (15.5)	55.4 (16.2)	69.6 (14.0)	15.12 (4.97)**	14.88 (4.93)**	1.43 (3.15)
Others	60.5 (17.2)	62.2 (15.1)	73.8 (10.9)	15.05 (3.69)***	12.76 (3.34)***	2.13 (2.84)

*Model with random intercept for age, gender and no use of CP, CP in development or CP

** P<0.05

*** P<0.001

NCP: no care pathway

CP: care pathway

SD: standard deviation

SE: standard error

### Interclass correlation coefficient (ICC)

ICCs were calculated at the team level (n = 144) for care processes organized by CP or NCP (Table 3). For both CP and NCP, the ICCs suggest that across teams there was high agreement about the quality of care processes. For the overall CPSET and all its subscales, except follow-up of care, agreement was lower for CP compared to NPC. The ICCs for CP ranged from 0.098 for collaboration with primary care to 0.216 for coordination of care. For NCP the ICC ranged from 0.162 for collaboration with primary care, to 0.256 for communication with patient and family.

**Table 3:** ICCs and 95% CI for care processes organized by NCP and CP.

Scores at team level	CP (n = 46)		NCP (n = 98)	
	ICC	95% CI	ICC	95% CI
Overall CPSET score	0.196	0.138–0.254	0.235	0.151–0.318
Sub-scales				
Patient focused organization	0.178	0.123–0.233	0.219	0.138–0.300
Coordination of care	0.216	0.156–0.277	0.245	0.160–0.330
Communication with patient and family	0.171	0.117–0.225	0.256	0.169–0.343
Collaboration with primary care	0.098	0.056–0.140	0.162	0.092–0.232
Follow-up of care	0.213	0.153–0.273	0.170	0.099–0.241

NCP: no care pathway

CP: care pathway

ICC: interclass correlation coefficient

95% CI: 95% confidence interval

### Predicting well-organized care processes

Sensitivity, specificity and the odds ratio (OR) for care processes organized with or without CP are shown in Table 4. For the overall CPSET score, care processes with CP enabled us to identify 74% of the well-organized care processes (sensitivity). Forty-seven percent of the weakly organized care processes had NCP (specificity). Care process with implemented care pathways are 2.6 times more likely to be well organized.

**Table 4:** Sensitivity and specificity analysis for care processes organized by NCP and CP.

Scores	Sensitivity*(%)	Specificity*(%)	OR	95% CI
Overall CPSET score	0.739 [0.705–0.771]	0.474 [0.444–0.505]	2.556	2.071–3.155
Sub-scales				
Patient focused organization	0.844 [0.815–0.869]	0.234 [0.209–0.261]	1.653	1.286–2.125
Coordination of care	0.806 [0.775–0.834]	0.346 [0.318–0.376]	2.208	1.756–2.272
Communication with patient and family	0.573 [0.536–0.609]	0.539 [0.508–0.569]	1.567	1.290–1.903
Collaboration with primary care	0.627 [0.590–0.662]	0.451 [0.421–0.482]	1.381	1.133–1.684
Follow-up of care	0.692 [0.657–0.725]	0.608 [0.578–0.638]	3.494	2.848–4.286

*The cut off value is 0.65

OR: odds ratio

95% CI: 95% confidence interval

## Discussion

The implementation of a CP is related to better organization of the care process, as measured by the overall CPSET score, and the subscales coordination of care and follow-up of care. The highest effect was noticed for follow-up of care. No improvements in scores, on the different subscales, were seen when NCP was compared to CP in development. The variations in mean scores were reduced for the overall CPSET scales and the five subscales when a CP was implemented. This was also the case for the ICCs. In almost 75% of the cases when a CP was used, this led to a well-organized care process but in almost half of the cases the care process was well-organized compared with NCP. Based on the cut off scores, identified in the study by Seys et al., the average scores of care processes with CP can be found in a higher percentile for the subscales follow-up of care, communication with patient and family, coordination of care, patient-focused organization and the overall CPSET score [25]. So CP have a strong impact on the organization of care.

Limitations of this study should be taken into consideration. First, the CPSET tool itself is based on self-evaluation based on the perception of healthcare professionals which can lead to bias. Based on the high ICC’s a multilevel model was used. Age and gender were used as covariates in our model. But no correction was made for professional group, as we wanted to know the impact of the different healthcare professional on the organization of care. The results of this study might be biased by using a convenience sample and the fact that the involved organizations are member of the Belgian-Dutch Clinical Pathway Network. The management of the hospital makes the decision to participate in this Network. These organizations are interested in improving the organization of care and have a positive attitude regarding working with care pathways. In general the effect of the organization of care might be higher in both groups compared to no network hospitals.

The results of a previous study by Vanhaecht et al. (2009) showed no significant differences on the subscales patient focused organization, communication with patient and family and collaboration with primary care in care process with CPs and NCPs [22]. Five years later, we can conclude that organizations should still pay attention to coordination and communication because these are identified as the most important priorities for patient safety research in developed countries [28] and had still the lowest performance rates after implementation of a CP. Communication is, in our study, included in the subscale communication with patient and family but communication is also included in all the five subscales. As a CP has a significant effect on the overall CPSET, coordination of care and follow-up of care, CPs can be used as an intervention to improve communication and can have an impact on patient safety. The link between CPs and patient safety was found earlier. A Cochrane review showed that CPs are associated with improved documentation and reduced in-hospital complications [15]. On the other hand, communication should be standardly included in the basic training of all healthcare professionals. Because poor communication among healthcare professionals is seen as a contributing factor in more than half of the adverse events [29].

The ICC in our study were high and the range was smaller in CP compared to NCP. A multilevel analysis was performed at team level. The subscale collaboration with care (3 items) has the lowest ICC which means that there is more variability, due to the fact that the scores of the different teams correlate less to each other. The results show that there is more agreement in care process organized by NCP than CP, showing less variability in NCP teams. We expected the opposite because healthcare professionals are normally more involved in the care process and are better organized which would lead to higher scores and more agreement between team members [25]. On the other hand, in a care process organized by NCP on average 10 team members were involved, while for CP this was 15 team members. More team members mean higher change of different perceptions of the involved healthcare professionals about the organization of care, leading to more variability in perception between team members.

An evolution in CPSET scores is observed. Our study had lower scores on all the subscales for care processes with NCP, CP under development and CP compared with the scores on the subscales mentioned in the previous study of Vanhaecht et al. [22]. That is also the explanation why the cut-off scores for the sensitivity and specificity analysis is 0.65, compared with 0.70 in the study of Vanhaecht et al. [22]. The lower scores mean that healthcare professionals perceive the organization of care as less organized. A possible explanation could be found in that healthcare professionals are more critical against the organization of their care process. They realize that quality and quality improvement projects are important, have more experience with developing and implementing of CP and are trying to imbed them in the organization culture. Also, in this study we pursued CPSET scores with a different study design. Here, these scores were calculated from all team members, whereas previously these were calculated from one medical doctor, one head nurse and one pathway facilitator [22].

Our study showed that care pathways had an effect on the perception of healthcare professionals about the organization of care processes. Physicians perceived the quality of organized care as highest compared to the other healthcare professionals. A recent publication showed a positive significant effect for the implementation of a care pathway on team input indicators, e.g. work environment, team composition, but also on conflict management and team climate for innovation but also lead to an increase in the organizational level of care processes. The risk for burnout decreased with a significant lower score on emotional exhaustion and a significant increase in the level of competence [18]. High-performance teams is defined by Kozlowski as “a multilevel process that results from team members’ engagement to accomplish individual-level and team-level taskwork and teamwork” [18]. For the development and implementation of care pathways it is important for teams to be involved in the whole process and support the project, as part of the Goals-Roles-Processes-Interprofessional Relations (GPRI) model, in this way high-performing teams can be built [18].

This study and other previous studies showed that care pathways have an impact on the organization of care, and an effect on team and on patient outcomes [15, 18, 23]. Further research is needed to find a relationship between these three elements. Does this mean that when a care pathway is used patients perceive the organization of care to be better? Which are the success factors for the implementation of care pathways perceived by patients and healthcare professionals? Care pathways should be embedded in the culture and the policy of the organization but which factors are leading to sustain a care pathway? Our results show that CP leads to better organized care processes based on a mix of care processes but does this also lead to less variation in teams?

## Conclusion

Care pathways have a positive impact on the organization of care processes, but not all CP are well-organized. By using a CP the care process is more coordinated and better followed up. Managers and teams should use CP as a way to increase the quality of their care process organization and make healthcare professionals become more aware of their role in the organization of the process. By using CPSET on regular time points, managers and care pathway facilitators can take actions when needed to improve the actual organization of the care process.

## Supporting information

S1 Table The Care Process Self-Evaluation Tool (CPSET) is a tool to measure the perception of team members concerning the organization of the care process. (PDF)

## Abbreviations

95% CI: 95% confidence interval
CP: Care pathway
CPSET: Care Process Self-Evaluation Tool
ICC: Interclass correlation coefficient
NCP: No care pathway
OR: Odds Ratio
SD: Standard deviation
SE: Standard error
