# Supplementary material for: Better organized care via care pathways: A multicenter study
Source: PLoS One. 2017 Jul 3;12(7):e0180398. doi: 10.1371/journal.pone.0180398 (PMC5495424; doi:10.1371/journal.pone.0180398)
Supplement: S1 Table — (PDF) [file pone.0180398.s001.pdf]

KU LEUVEN
LEUVEN INSTITUTE FOR HEALTHCARE POLICY

S1 Table

How do you agree with the following statements? Strongly disagree(1) Strongly agree(10)

**Patient focused organization**		**1**	**2**	**3**	**4**	**5**	**6**	**7**	**8**	**9**	**10**
PO1	A patient focused vision exists within the organization.										
PO2	Quality of care is the priority within the organization.										
PO3	The care process coordinator has a patient focused vision.										
PO4	Patient communication is considered to be important within the organization.										
PO5	The organizational structure is patient focused.										
PO6	There is a clear vision of policy regarding care throughout the entire hospital.										
**Coordination of the care process.**		**1**	**2**	**3**	**4**	**5**	**6**	**7**	**8**	**9**	**10**
COR1	Agreements are observed.										
COR2	All team members are familiar with the various steps in the care process.										
COR3	There is an optimum timing of activities within the care process.										
COR4	Concrete agreements are made within the care process.										
COR5	Team members consider themselves to be engaged in the organization of the care process.										
COR6	Patients/family are provided with candid (frank, open; straightforward) information regarding their health.										
COR7	Discharge is communicated in a timely manner to the patient and family so that they can take necessary measures.										
**Communication with patient and family**		**1**	**2**	**3**	**4**	**5**	**6**	**7**	**8**	**9**	**10**
COM1	Within the care process time is explicitly provided to listen to the patient and his family.										
COM2	Time is explicitly scheduled within the care process for communications between healthcare professional and patient.										
COM3	Within the care process there is provision for sufficient time to provide information.										
COM4	The patient is explicitly asked for his consent with regard to the proposed care.										
**Collaboration with primary care.**		**1**	**2**	**3**	**4**	**5**	**6**	**7**	**8**	**9**	**10**
SE1	Primary care is considered by the hospital to be an equal partner.										
SE2	Good cooperation exists between the hospital and primary care.										
SE3	In complex care situations consultation takes place between the physician/surgeon and general practitioner.										
**Monitoring and follow-up of care process**		**1**	**2**	**3**	**4**	**5**	**6**	**7**	**8**	**9**	**10**
OP1	When (re)designing the care process quality indicators are formulated.										
OP2	Whether the care provided is tailored to the patient's needs is systematically monitored/followed-up.										
OP3	Within the care process patient satisfaction is monitored/followed-up systematically.										
OP4	The goals of the care process are described explicitly.										
OP5	Within the care process monitoring/follow-up is performed to verify whether planned activities are actually performed.										
OP6	Outcomes are systematically monitored/followed-up.										
OP7	Variances can be monitored within the care process.										
OP8	Within the care process risks of complications are monitored / followed-up systematically.										
OP9	The progress in the care process is continuously monitored/followed-up and adjusted.										

© Leuven Institute for Healthcare Policy
